# Integrated community case management plus compared with outpatient therapeutic programs: a quasi-experimental study among children with severe acute malnutrition in Jowhar, Somalia

**DOI:** 10.1016/j.ajcnut.2026.101391

**Published:** 2026-06-09

**Authors:** Iris Bollemeijer, Jennifer Majer, Naomi Mwikali Ndung’u, Zamzam Daud Ahmed, Ahmed Elyas Mohamud, Gulled Abdi Gulled, Hans Verhoef, Alida Melse-Boonstra, Sarah King

**Affiliations:** 1 International Medical Corps, Los Angeles, CA, United States; 2 Wageningen University and Research, Division of Human Nutrition and Health, Wageningen, The Netherlands; 3 International Medical Corps, Mogadishu, Somalia; 4 US Centers for Disease Control and Prevention, Department of Health and Human Services (HHS), Atlanta, GA, United States

**Keywords:** iCCM+, severe acute malnutrition, outpatient therapeutic program, community health workers, community management of acute malnutrition, Somalia

## Abstract

**Background:**

WHO guidelines endorse treatment of severe acute malnutrition (SAM) by community health workers as an alternative to facility-based care, but supportive evidence for this shift is low.

**Objectives:**

Among Somali children with uncomplicated SAM, we compared recovery between facility-based outpatient therapeutic programs (OTP) and integrated community case management (iCCM+), examining weight change, length of stay, discharge anthropometry, and overall anthropometric change.

**Methods:**

In a quasi-experimental study (3 January–19 May, 2024), children with uncomplicated SAM [weight-for-height Z-scores (WHZ) < –3 SD and/or mid-upper arm circumference (MUAC) ≤115 mm] were enrolled for treatment in OTP (2 sites, 638 children) or iCCM+ (8 villages, 545 children). During weekly follow-up visits, children received nutritional assessments, ready-to-use therapeutic food, and routine medications until discharge (MUAC >115mm; WHZ > –3SD). Weight change and time-to-recovery were analyzed using generalized linear mixed-effects models and Cox proportional hazards models, respectively. To adjust for baseline differences in observed covariates, we used propensity score adjustment.

**Results:**

At enrolment, OTP children had more severe anthropometric deficits than their peers in the iCCM+ group (WHZ<–3SD: 48% compared with 42%, *P* = 0.04; MUAC ≤110 mm: 34% compared with 18%, *P* < 0.001). Recovery proportions were similar between groups (iCCM+: 89%; OTP: 91%). In adjusted analyses, no difference was observed in weight change over time. The iCCM+ group had a shorter median length of stay (by 7 d) and faster time-to-recovery (adjusted hazard ratio: 3.32, 95% CI: 2.70, 4.09).

**Conclusions:**

Treatment outcomes were similar between approaches; iCCM+ enabled earlier identification and shorter length of stays, suggesting improved efficacy and reduced burden on caregivers and health systems. It shows promise for expanding SAM treatment in settings with limited OTP access.

## Introduction

Community health workers (CHWs) are increasingly involved in the detection and treatment of severe acute malnutrition (SAM), which has traditionally been managed at clinics through the community-based management of acute malnutrition (CMAM) model. CMAM requires regular clinic visits, creating access barriers [1,2]. To extend coverage of services, SAM treatment is increasingly piloted through community-based platforms, including Integrated Community Case Management (iCCM) which provides treatment for childhood illnesses including malaria, pneumonia, and diarrhea. Integration of SAM care into iCCM, known as iCCM+, allows CHWs to deliver care at the household while also addressing these common childhood illnesses. This integrated model holds promise for improving coverage, promoting early detection, and reducing defaulting in hard-to-reach settings [3].

The need to expand access to SAM treatment is urgent. In 2024, an estimated 12.2 million children aged <5 y were affected by SAM [4], yet only 7.3 million received treatment [5]. The UN Global Action Plan for Wasting aimed to increase coverage by 50% by 2025 [6], but progress has been constrained by fragile health systems, workforce gaps, and limited funding. These persistent gaps have increased interest in alternative service delivery models that can bring treatment closer to affected communities.

Evidence to date suggests that CHW-led SAM treatment can achieve outcomes comparable to facility-based CMAM, with fewer defaulters and higher coverage [[7], [8], [9]]. Early findings also indicate potential cost savings [[10], [11], [12]]. The 2023 WHO wasting guidelines endorse CHW-led SAM treatment on the condition of adequate monitoring and training, but cite low evidence certainty, underscoring the need for further research on iCCM+ [13]. Important evidence gaps remain, particularly regarding implementation in complex humanitarian settings with limited human and material resources [8]. To the best of our knowledge, treatment progression and outcomes under this approach have not been studied in the fragile humanitarian context of Somalia.

This study compared outcomes and treatment progression among children with SAM managed through an iCCM+ approach compared with outpatient therapeutic programs (OTP). The study aimed to provide context-specific evidence to inform national policy and guidelines and programmatic decisions on expanding iCCM+ in Somalia.

## Methods

### Study setting and staffing

This study was a quasi-experimental, nonrandomized comparative effectiveness study conducted in Jowhar District, in Somalia, where the global acute malnutrition and SAM prevalence was 17.5% and 3.5%, respectively [14]. In 2024, International Medical Corps (IMC) supported 22 OTP facilities and 9 iCCM+ sites in Jowhar District. Two health facilities (supported by IMC since 2018 onward) and 8 villages (supported by IMC since 2024 onward) were purposively selected based on treatment consistency (uninterrupted services and protocol compliance), balanced caseloads (case enrolment similar across sites), community representation (geographic diversity), and physical accessibility for both staff and community. A total of 16 iCCM+ staff were employed (2 per village), whereas 5 OTP staff were employed at the 2 health facilities. iCCM+ staff were purposively selected and were newly recruited based on residency in the targeted village, literacy and education level, and previous work experience. Half of the iCCM+ staff completed secondary education or higher, and those who did not complete secondary education were all literate. Only 2 iCCM+ staff had <1 y of work experience, whereas all others had >1 y of working experience. OTP staff were already employed in the 2 OTP sites at the start of the study and were registered nurses (4 OTP staff) or registered first aid responders (1 OTP staff). All OTP staff had between 3 and 5 y of work experience. Both ICCM+ and OTP staff received a 6-d training on SAM management using Somali Ministry of Health (MOH) training material. Continuous on-the-job training and joint supervision were provided, which was more intensive at the start, with 3 visits per week, and was reduced to twice per week after the first month of implementation. A forthcoming paper will present the accuracy of nutrition status classification between the iCCM+ and OTP staff and will discuss operational aspects in more detail.

### Program description

Children were screened for acute malnutrition (AM) using mid-upper arm circumference (MUAC), weight-for-height z-score (WHZ), and bilateral pitting edema. In iCCM+ villages, CHWs conducted screenings, whereas in OTP catchment areas, children were screened through mass MUAC screenings and referred to CMAM if required. Uncomplicated SAM was defined per WHO guidelines [8] and the Somali national protocol, as MUAC <115 mm, a WHZ <–3 SD, and/or bilateral pitting edema without medical complications [15].

Treatment in both groups included weekly visits during which ready-to-use therapeutic food (RUTF), routine medication (age-based dose of amoxicillin and albendazole), and nutrition counseling were provided. Children received a weight-based dose of RUTF, the equivalent to about 170 kcal/kg/d. OTP visits took place at static facilities, whereas iCCM+ visits occurred at households of children identified with uncomplicated SAM. iCCM+ collected the required weekly supplies from nearby primary health units where IMC provided basic health care services by hired motorbikes. Children were discharged on reaching moderate AM criteria (MUAC > 115 - ≤ 125mm and WHZ > -3 - ≤ -2 SD, and no edema for 2 consecutive visits) or for defaulting (missing 2 consecutive visits), transferring (medical complications or relocation), nonresponse (failure to meet discharge criteria after 12 wk), or death.

### Sample size

Sample size was calculated to detect a 10% difference in the proportion recovered between iCCM+ and OTP. On the basis of prior evidence and program data, the recovery proportion was estimated at 80%. Sample size calculations were performed in Stata 17 using the following assumptions: 80% power and a 0.05 *α* level; a 2-sided comparison; a design effect of 1.5 to account for population variability across sites [16]; and 10% loss to follow-up. The minimum required sample size per group was 332.

### Data collection

Children were enrolled on a rolling basis between 3 January and 19 May, 2024. Eligibility was restricted to children aged 6 to 48 mo because a second phase of the study was planned to assess sustained recovery 12 mo after discharge. Restricting enrolment to children aged ≤48 months ensured that participants would remain within the <5 year age range at follow-up and that anthropometric criteria would remain appropriate and comparable over time. Exclusion criteria included prior inpatient SAM treatment, chronic or congenital conditions affecting growth or nutritional intake, and residence outside the study area. Caregivers of eligible children were read a consent script by study staff explaining the purpose of the study and their right to refuse participation. Because of low literacy, verbal rather than written informed consent was obtained and recorded before enrolment and at each follow-up visit.

Baseline child and household characteristics were collected during enrolment using standardized forms. Variables included, but were not limited to, self-reported child’s age and sex, child feeding, caregiver’s age, household hunger score, household income, maternal education level, displacement status, household size, and access to assistance. Data were captured at admission, weekly follow-up through discharge and included anthropometrics (weight, height, MUAC, and edema), episodes of malaria, diarrhea, and pneumonia; and quantity of RUTF provided. iCCM+ and OTP staff recorded data using structured paper forms and registers, which were entered by data clerks into an electronic format, Ona [17]. Quality checks were conducted weekly during data entry by research supervisors, and any errors were corrected by referencing the original paper forms.^.^

### Outcomes

The primary outcomes were treatment outcomes including recovery. Secondary outcomes included length of stay (LoS), cumulative RUTF received, anthropometric status at discharge (MUAC, WHZ, weight for age Z-score, and height for age Z-score), and anthropometric change during treatment, including total weight gain, weight gain per day, total MUAC gain, and MUAC gain per day. Change in weight over time was assessed using repeated weekly weight measurements collected during follow-up. Time-to-recovery was defined as the number of days from admission to recovery.

### Statistical analysis

Data were exported from Ona and cleaned in Stata 17 [18]. All anthropometric indices were computed using WHO 2006 Child Growth Standards, Stata package *zscore06* [19]. Proximal linear interpolation addressed missing or implausible anthropometric indices between admission and discharge [20]. T-tests compared means between study groups and between interpolated and noninterpolated values within cohorts.

Children were excluded from analyses if age at admission fell outside of the eligible 6 to 48 months criteria, admission or discharge anthropometrics were implausible per WHO flags [21], LoS exceeded 120 days, dates of admission and/or discharge were missing, or age at enrolment was missing. Children who remained in treatment at the end of the study were censored and included in time-to-event analyses, but excluded from discharge-based analyses requiring a confirmed treatment outcome.

Baseline characteristics were summarized using unweighted descriptive statistics. Results are presented as frequencies and percentages for categorical variables and as means (SD) or medians (IQR) for continuous variables. Between-group differences in baseline characteristics were assessed using Pearson’s chi-square test or Fisher’s exact tests for binary and categorical variables. For continuous variables, t-test was applied to compare means, or the Mann–Whitney U test was applied to compare distributions.

Given the nonrandomized design, we employed propensity scores to reduce possible selection bias. Scores were estimated through a logistic regression model that included baseline variables predictive of recovery: age, sex, and anthropometrics at admission (MUAC and WHZ), breastfeeding status [22], household hunger scale [23], nutrition assistance received [24] and maternal education [25]. The probability of enrolment in iCCM+ was predicted for each child enrolled, and the resulting propensity score was included as a covariate in adjusted models to account for observed baseline differences between groups. Scores were normally distributed (Supplemental Figure 1).

Change in weight over time during treatment was analyzed using a generalized linear mixed-effects model with repeated child weight measurements at weekly follow-up visits as the dependent variable. The model included a random intercept at the participant level to capture individual variability and adjusted for fixed baseline covariates: treatment site, child age, sex, mean-centered MUAC at admission, mean-centered WHZ at admission, and the propensity score. To assess whether weight changed differently over time by study group, the model included an interaction between study group and visit week. Additional interaction terms were included for visit week by admission WHZ and study group by admission WHZ. Continuous covariates were mean centered.

Time-to-recovery was assessed using a Cox proportional hazards model with time defined as days from admission to recovery. Children who remained in treatment at the study end date were censored at their last observed follow-up visit. The model included study group, child age, sex, MUAC at admission, WHZ at admission, and propensity score as covariates. Cluster-robust SEs were specified at the child level. Analyses were conducted on an intention-to-treat basis. Per-protocol, defined as children who completed treatment and were discharged as recovered, estimates are provided as supplementary materials (Supplemental Table 1).

Analyses were conducted using R version 4.3.2 [26] and R Studio (2024.12.1+563) [27]. The following packages were used; *dplyr* and *gtsummary* [28] for descriptive statistics, *ggplot2* [29] for charts, *survival* for Cox models, *forestplot* [30] for forest plots, and *lme4* [31] for mixed effects models.

### Ethical review and registration

Institutional research board (IRB) approval was obtained from the Somali Research and Ethical Committee at the Ministry of Health on 22 November, 2023 (Ref/MOHHS/DGO/0846/Nov2023). The study was reviewed by the United States Centers for Disease Control and Prevention (CDC) and was conducted consistent with applicable federal law and CDC policy. The study was retrospectively registered under registration number PACTR202509657291953.

## Results

**FIGURE 1:**
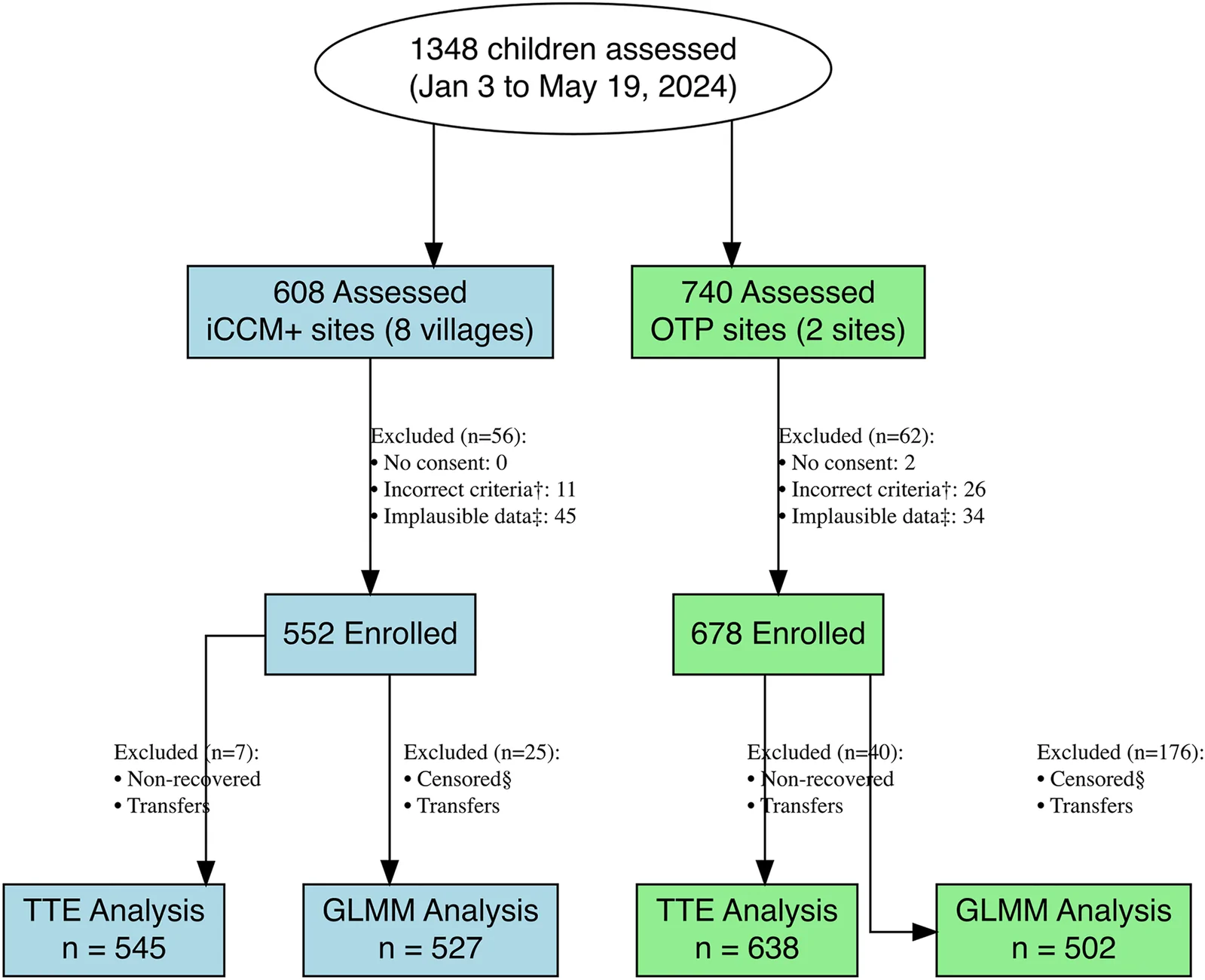
Participant flow through a quasi-experimental study comparing treatment outcomes of severely acutely malnourished children aged 6 to 48 mo at community health (iCCM+) sites (*n* = 8 villages) and OTP sites (*n* = 2 health facilities), Jowhar District, Somalia, January–May 2024. Children were enrolled on a rolling basis and followed weekly until discharge. Exclusions are shown at each stage; totals represent unique children, and listed reasons are not mutually exclusive. Children still in treatment at the study end date were censored and included in time-to-event analyses only. Analytical subsets for the intention-to-treat GLMM and TTE analyses are indicated. GLMM, general linear mixed models’ analysis; HAZ, height for age Z-score; iCCM+, integrated community case management; MUAC, mid-upper arm circumference; OTP, outpatient treatment program; SAM, severe acute malnutrition; TTE, time-to-event; WHZ, weight-for-height Z-score. † Incorrect criteria = age <6 mo or age >48 mo at admission and/or not SAM at admission. ‡ Implausible data = anthropometric data (MUAC and/or WHZ/HAZ) outside plausible range at admission/discharge. § Censored = children still enrolled in treatment at study end date.

One thousand three hundred forty-eight children were assessed for eligibility after admission for malnutrition treatment, among whom 608 were eligible at iCCM+ sites and 740 were eligible at OTP sites (Figure 1). A total of 118 unique children were excluded from analyses; recorded exclusion reasons, which were not mutually exclusive, included failure to meet predefined eligibility criteria in 37 children (OTP: 26, iCCM+: 11) and missing or implausible values in 81 instances (OTP: 34, iCCM+: 45). The overall proportion excluded was similar across groups (9.2% in iCCM+ and 8.4% in OTP). Outcomes for 32 children were reclassified after recalculation of nutrition status at discharge. At the end of the study, 154 children remained enrolled in treatment and were censored in the relevant models.

Among children included in the final analysis, 88% attended all follow-up visits (Supplemental Table 2). Missed visits were more common in OTP than iCCM+ (20% compared with 2%). Only 5% of anthropometric measurements were missing. Interpolated values were comparable to the original data, with no difference in mean values (Supplemental Table 3)**.**

### Admissions

Baseline household characteristics were largely similar between groups (Table 1). Mean age was 16.3 months (median: 12.1) in iCCM+ and 17.5 months (median: 14.0) in OTP. Children in OTP were more likely to be female (60% compared with 52%, *P* = 0.009) and were less likely to be currently breastfed (60% compared with 68%, *P* = 0.003). Vaccination was higher in OTP (98% compared with 76%, *P* < 0.001), and a greater proportion of caregivers reported seeking health care services when a child was ill (98% compared with 86%, *P* < 0.001).

**TABLE 1:** Baseline household and child characteristics at admission among children aged 6–48 mo enrolled for treatment of SAM at iCCM+ (*n* = 545) and OTP (*n* = 638) sites, Jowhar District, Somalia, January–May 2024

Variable	*N*	Overall *N* = 1183^1^	ICCM site *N* = 545^1^	OTP site *N* = 638^1^	*P* value^2^
Household members (*n*)	1183	7 (6, 9)	7 (5, 8)	7.00 (6, 9)	<0.001
Household members aged <5 y (*n*)	1183	2 (2, 3)	2 (2, 3)	3.00 (2, 3)	<0.001
Primary source of income	1183				<0.001
Animal husbandry		41 (3%)	35 (6%)	6 (1%)	
Casual labor		471 (40%)	91 (17%)	380 (60%)	
Farming		592 (50%)	407 (75%)	185 (29%)	
None or other		79 (7%)	12 (2%)	67 (11%)	
Displacement status	1177				0.058
Internally displaced		7 (1%)	5 (1%)	2 (0%)	
Never displaced		1142 (97%)	520 (96%)	622 (98%)	
Returnee		28 (2%)	18 (3%)	10 (2%)	
Unknown		6	2	4	
Mother’s education	1182				0.047
No education		1132 (96%)	530 (97%)	602 (95%)	
Primary education		8 (1%)	1 (0%)	7 (1%)	
Secondary education and up		2 (0%)	0 (0%)	2 (0%)	
Some primary education		40 (3%)	14 (3%)	26 (4%)	
Unknown		1	0	1	
Primary caregiver	1180				0.058
Father		7 (1%)	5 (1%)	2 (0%)	
Grandmother		7 (1%)	6 (1%)	1 (0%)	
Mother		1160 (98%)	529 (97%)	631 (99%)	
Sibling		6 (1%)	4 (1%)	2 (0%)	
Unknown		3	1	2	
Mother alive	1178	1169 (99%)	537 (99%)	632 (100%)	0.090
Unknown		5	1	4	
Father alive	1177	1167 (99%)	536 (99%)	631 (99%)	0.527
Unknown		6	3	3	
Mother’s age	1181	28 (24, 31)	27 (23, 32)	28 (25, 30)	0.346
Unknown		2	0	2	
Household Hunger Scale	1158				<0.001
Little to no hunger		411 (35%)	213 (40%)	198 (32%)	
Moderate hunger		458 (40%)	162 (31%)	296 (47%)	
Severe hunger		289 (25%)	156 (29%)	133 (21%)	
Unknown		25	14	11	
Child age at admission (mo)	1183	13 (9, 24)	12 (9, 21)	14 (10, 24)	0.041
Child sex	1183				0.009
Female		669 (57%)	286 (52%)	383 (60%)	
Male		514 (43%)	259 (48%)	255 (40%)	
Child is a twin	1182	34 (3%)	16 (3%)	18 (3%)	0.902
Unknown		1	1	0	
Humanitarian assistance	1183	77 (7%)	64 (12%)	13 (2%)	<0.001
Food aid	1183				0.184
No		1167 (99%)	535 (98%)	632 (99%)	
Yes, received in-kind food aid		16 (1%)	10 (2%)	6 (1%)	
Food voucher support	1183				0.101
No		1177 (99%)	540 (99%)	637 (100%)	
Yes, received food voucher		6 (1%)	5 (1%)	1 (0%)	
Cash support	1183				<0.001
No		1146 (97%)	508 (93%)	638 (100%)	
Yes, received cash assistance		37 (3%)	37 (7%)	0 (0%)	
WASH services	1183				0.067
No		1167 (99%)	534 (98%)	633 (99%)	
Yes, received WASH support		16 (1%)	11 (2%)	5 (1%)	
Agriculture support	1183				>0.999
No		1180 (100%)	544 (100%)	636 (100%)	
Yes, received agriculture support		3 (0%)	1 (0%)	2 (0%)	
Admission criteria	1062				0.155
Admission by MUAC only (no edema)		552 (52%)	244 (54%)	308 (50%)	
Admission by edema (with or without other criteria)		26 (2%)	10 (2%)	16 (3%)	
Admission by WHZ and MUAC (no edema)		379 (36%)	162 (36%)	217 (35%)	
Admission by WHZ only (no edema)		105 (10%)	34 (8%)	71 (12%)	
Unknown		121	95	26	
MUAC at admission	1183	112 (110, 114)	112 (111, 114)	112 (110, 114)	0.002
MUAC category at admission	1183				<0.001
≤110 mm		317 (27%)	98 (18%)	219 (34%)	
>110 to ≤115 mm		767 (65%)	419 (77%)	348 (55%)	
>115 to ≤120 mm		74 (6%)	20 (4%)	54 (8%)	
> 120 mm		25 (2%)	8 (1%)	17 (3%)	
WHZ at admission	1183	−2.89 (−3.53, −2.07)	−2.80 (−3.46, −1.94)	−2.96 (−3.61, −2.18)	0.002
WHZ category at admission	1183				0.044
< −3 SD		539 (46%)	230 (42%)	309 (48%)	
−3 to −2 SD		370 (31%)	173 (32%)	197 (31%)	
> −2 SD		274 (23%)	142 (26%)	132 (21%)	
Edema at admission	1,063	26 (2%)	10 (2%)	16 (3%)	0.679
Unknown		120	94	26	
HAZ at admission	1183	−2.51 (−3.59, −1.18)	−2.59 (−3.71, −1.22)	−2.46 (−3.52, −1.15)	0.164
HAZ category at admission	1183				0.255
< −3 SD		454 (38%)	220 (40%)	234 (37%)	
−3 to −2 SD		270 (23%)	127 (23%)	143 (22%)	
> −2 SD		459 (39%)	198 (36%)	261 (41%)	
WAZ at admission	1183	−3.32 (−3.93, −2.51)	−3.25 (−3.95, −2.50)	−3.36 (−3.92, −2.57)	0.801
WAZ category at admission	1183				0.324
< −3 SD		707 (60%)	323 (59%)	384 (60%)	
−3 to −2 SD		333 (28%)	148 (27%)	185 (29%)	
> −2 SD		143 (12%)	74 (14%)	69 (11%)	
Child ever breastfed	1178	1171 (99%)	540 (99%)	631 (99%)	>0.999
Unknown		5	2	3	
Child currently breastfed	1183	755 (64%)	372 (68%)	383 (60%)	0.003
Child vaccinated	1183	1041 (88%)	415 (76%)	626 (98%)	<0.001
Child has diarrhea currently or within the last 7 d	1182	704 (60%)	320 (59%)	384 (60%)	0.634
Unknown		1	1	0	
Child has fever currently or within the last 7 d	1178	820 (70%)	364 (67%)	456 (72%)	0.076
Unknown		5	2	3	
Child has cough currently or within the last 7 d	1176	865 (74%)	387 (72%)	478 (75%)	0.176
Unknown		7	5	2	
Sought care for child’s last illness	1181	1090 (92%)	467 (86%)	623 (98%)	<0.001
Unknown		2	2	0	
Location of care seeking	1090				<0.001
Community health worker		388 (36%)	215 (46%)	173 (28%)	
Government facility		555 (51%)	152 (33%)	403 (65%)	
Mobile/outreach clinic		86 (8%)	59 (13%)	27 (4%)	
Private or other		61 (6%)	41 (9%)	20 (3%)	
Unknown		93	78	15	

Values are unweighted, unadjusted descriptive statistics. Continuous variables are presented as median (Q1, Q3); categorical variables are presented as *n* (%). *P* values were calculated using the Wilcoxon rank-sum test for continuous variables and Pearson’s chi-squared or Fisher’s exact test for categorical variables.

Abbreviations: HAZ, height for age Z-score; ICCM+, integrated community case management; MUAC, mid-upper arm circumference; OTP, outpatient therapeutic program; SAM, severe acute malnutrition; WASH, water, sanitation, hygiene; WAZ, weight for age Z-score; WHZ, weight-for-height Z-score.

1 Median (Q1, Q3); *n* (%).

2 Wilcoxon rank-sum test; Pearson’s chi-squared test; Fisher’s exact test.

Median MUAC and WHZ at admission were similar among both study groups (112 mm in both groups; –2.80 compared with –2.96 Z-scores in iCCM+ and OTP, respectively). However, a larger proportion of children in OTP were in the lowest MUAC category ≤110 mm (34% compared with 18%, *P* < 0.001) and in the lowest WHZ category of < –3SD (48% compared with 42%, *P* = 0.04). Admission criteria were similar across groups: 52% of children were admitted based on MUAC alone, WHZ-only admissions accounted for 10%, and 36% were admitted by both WHZ and MUAC.

### Treatment progress and discharge

**FIGURE 2:**
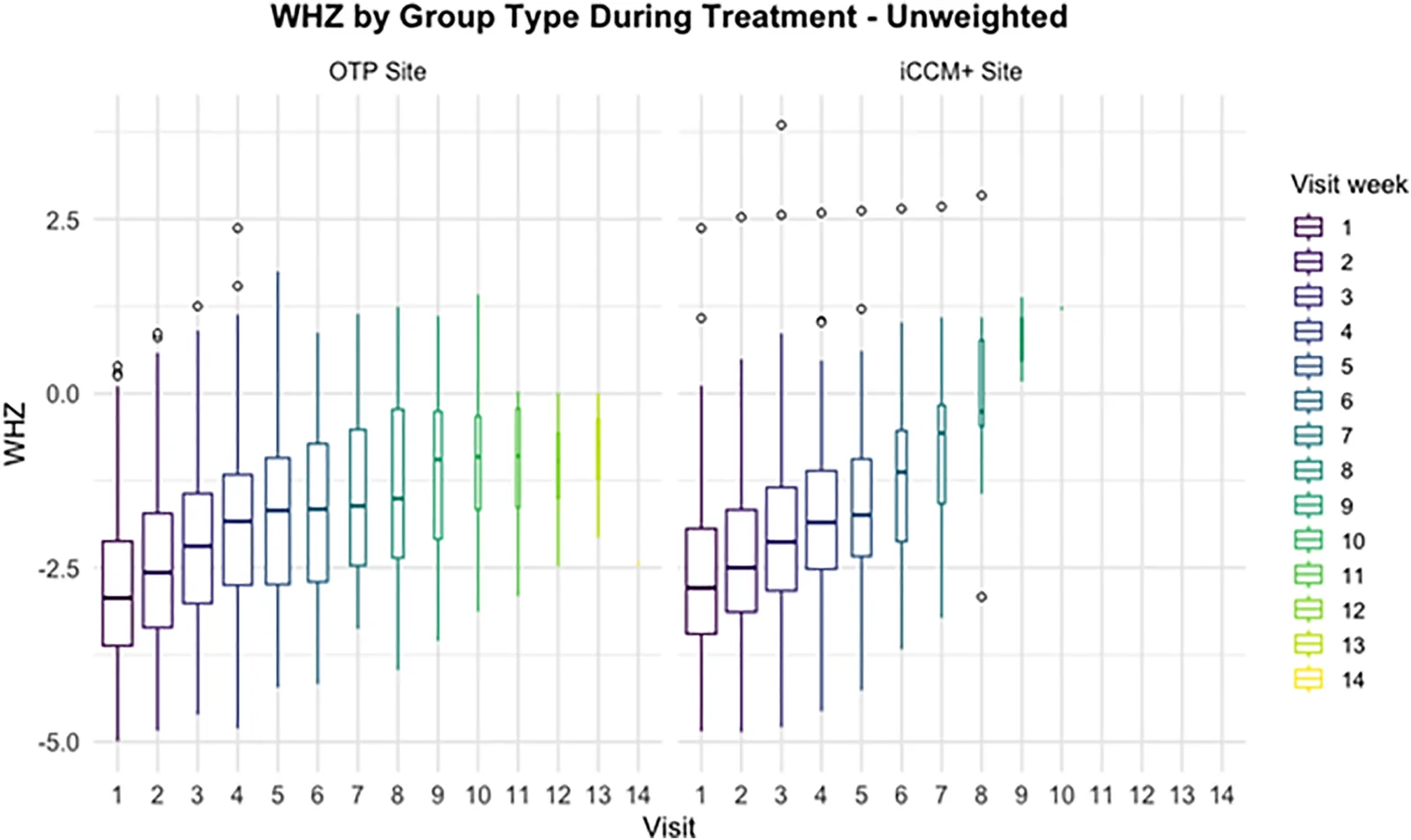
Mean WHZ by treatment visit week among children with severe acute malnutrition enrolled in iCCM+ and OTP groups, Jowhar District, Somalia, January–May 2024. Points represent mean WHZ at each weekly visit; error bars represent ± 1 SE. Box widths represent the number of children observed at each visit. Visit week 1 represents admission measurements. iCCM+, integrated community case management; OTP, outpatient therapeutic program; WHZ, weight-for-height z-score.

**FIGURE 3:**
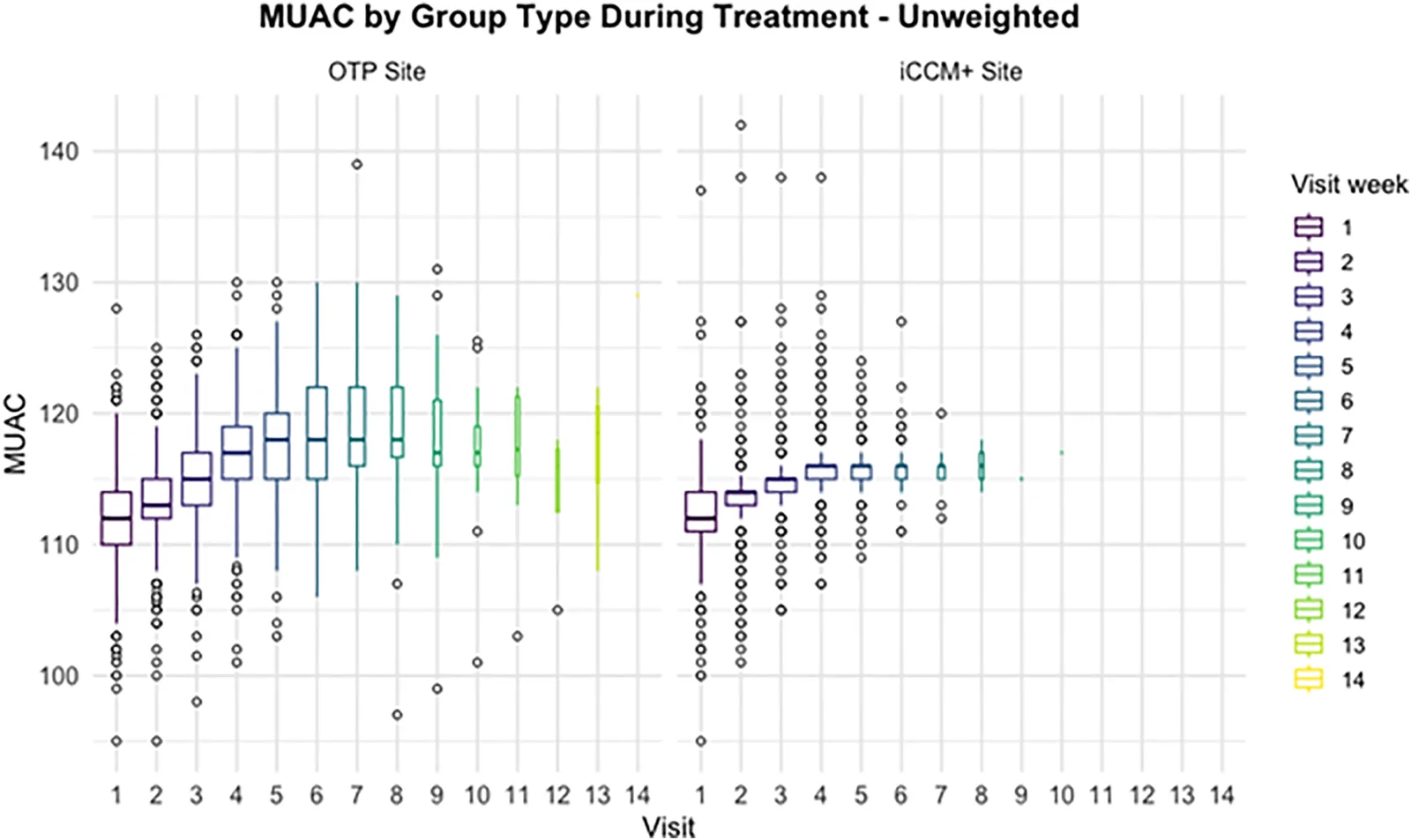
Mean MUAC (mm) by treatment visit week among children with severe acute malnutrition enrolled in iCCM+ and OTP groups, Jowhar District, Somalia, January–May 2024. Points represent mean MUAC at each weekly visit; error bars represent ± 1 SE. Box widths represent the number of children observed at each visit. Visit week 1 represents admission measurements. iCCM+, integrated community case management; MUAC, mid-upper arm circumference; OTP, outpatient therapeutic program.

Recovery was comparable between the 2 groups (ICCM+ 89% compared with OTP 91%, *P* = 0.3) (Table 2). No deaths or defaulters were observed in either group. LoS was shorter in iCCM+ compared with the OTP group (21 compared with 28 days, *P* < 0.001). The median weight gain was higher in OTP (0.8 kg compared with 0.6 kg, *P* < 0.001), as was the median MUAC gain (7.0 mm compared with 4.0 mm, *P* < 0.001). Although the anthropometric measurements among children discharged in the iCCM+ group were worse than those in the OTP group, all children were discharged with a MUAC and WHZ above the discharge criteria set by the Somali Nutrition cluster. Children admitted in the iCCM+ group had better anthropometric measures compared with children in the OTP group, limiting the comparison of overall weight and MUAC gain between groups. The worse anthropometric measurements at discharge among the iCCM+ group could potentially be explained by the fact that 11% of children in the iCCM+ group were discharged after reaching the discharge criteria during only 1 visit instead of 2 consecutive visits as per the intergrated managment of acute malnurition (IMAM) guidelines, whereas only 9% of children in OTP arm were discharged after reaching discharge criteria during 1 visit. Both MUAC and WHZ improved rapidly during early visits in both study groups, then plateaued with more gradual gains from the fourth visit onward, except for WHZ in the iCCM+ group, which continued to show rapid improvement throughout the course of treatment (FIGURE 2, FIGURE 3).

**TABLE 2:** Unadjusted, unweighted treatment outcomes and anthropometric changes at discharge among children with confirmed discharge outcomes enrolled in iCCM+ (*n* = 527) or OTP (*n* = 502) treatment for severe acute malnutrition, Jowhar District, Somalia, January–May 2024

Variable	*N*	Overall *N* = 1029^1^	ICCM site *N* = 527^1^	OTP site *N* = 502^1^	Difference (iCCM+ – OTP)	*P* value^2^
Recovered with ≥1 visit	1029	997 (97%)	526 (100%)	471 (94%)	6 pp	<0.001
Recovered with 2 consecutive visits	947	856 (90%)	460 (89%)	396 (91%)	−2 pp	0.308
Unknown		82	13	69		
Length of stay (d)	1009	28 (21, 32)	21 (21, 28)	28 (21, 40)	−7 d	<0.001
Unknown		20	0	20		
Cumulative RUTF received (# of sachets)	994	80 (65, 105)	80 (60, 95)	90 (75, 120)	−10 sachets	<0.001
Unknown		35	3	32		
WHZ at discharge	997	−1.61 (−2.35, −0.84)	−1.72 (−2.36, −0.96)	−1.46 (−2.29, −0.68)	−0.26 SD	0.003
Unknown		32	1	31		
Total weight gain (kg)	997	0.70 (0.50, 1.00)	0.60 (0.40, 0.80)	0.80 (0.60, 1.10)	−0.2 kg	<0.001
Unknown		32	1	31		
Weight gain per day (kg/d)	983	0.03 (0.02, 0.03)	0.02 (0.02, 0.03)	0.03 (0.02, 0.03)	0 kg/d	<0.001
Unknown		46	1	45		
MUAC at discharge (mm)	996	117 (116, 119)	116 (116, 117)	118 (117, 122)	−2 mm	<0.001
Unknown		33	1	32		
Total MUAC gain (mm)	996	5 (3, 8)	4 (3, 5)	7 (5, 10)	−3 mm	<0.001
Unknown		33	1	32		
MUAC gain per day (mm/d)	982	0.19 (0.14, 0.28)	0.14 (0.11, 0.20)	0.25 (0.20, 0.29)	−0.11 mm	<0.001
Unknown		47	1	46		
WAZ at discharge	997	−2.61 (−3.25, −1.88)	−2.71 (−3.36, −2.01)	−2.48 (−3.14, −1.75)	−0.23 SD	<0.001
Unknown		32	1	31		
HAZ at discharge	997	−2.87 (−3.94, −1.57)	−2.92 (−3.98, −1.58)	−2.85 (−3.91, −1.53)	−0.06 SD	0.634
Unknown		32	1	31		

Values are presented as *n* (%) for categorical outcomes and median (Q1, Q3) for continuous measures. The “Difference” column reports the unadjusted, unweighted difference in medians (continuous) or percentage points (categorical) between iCCM+ and OTP. Recovery is defined as reaching moderate acute malnutrition criteria (MUAC >115 mm, WHZ > −3 SD, and no edema) on ≥1 visit. Length of stay is calculated from admission to the date the discharge criteria were first met. *P* values are from Wilcoxon rank-sum tests (continuous) and Pearson’s chi-squared or Fisher’s exact tests (categorical). Adjusted comparisons for the primary outcomes are reported in the regression models.

Abbreviations: HAZ, height for age Z-score; ICCM+, integrated community case management; MUAC, mid-upper arm circumference; OTP, outpatient therapeutic program; pp, percentage points; RUTF, ready-to-use therapeutic food; WAZ, weight for age Z-score; WHZ, weight-for-height Z-score.

1 *n* (%); median (Q1, Q3).

2 Pearson’s chi-squared test; Wilcoxon rank-sum test.

### Weight gain

In the mixed-effects model (Table 3), there was no evidence of a difference in mean weight between study groups at the reference visit week [*β* = –0.01, 95% confidence interval (CI): –0.12, 0.10, *P* = 0.87]. There was also no evidence that the change in weight over time differed by study group, based on the interaction between study group and visit week (*β* = –0.01, 95% CI: –0.01, 0.00, *P* = 0.12), indicating no difference in weight gain trajectory.

**TABLE 3:** Fixed-effect estimates from a linear mixed-effects model with repeated weight measurements during treatment among children with severe acute malnutrition in iCCM+ and OTP groups, Jowhar District, Somalia, January–May 2024

Predictors	Mixed model treatment effect on weight during treatment	
	Estimates	CI
(Intercept)	7.30	7.16, 7.43
Study group (iCCM+)	−0.01	−0.12, 0.10
Visit week	0.18	0.18, 0.19
MUAC at admission	0.05	0.04, 0.07
WHZ at admission	0.34	0.27, 0.42
Child sex (male)	−0.10	−0.20, 0.01
Child age (mo)	0.15	0.14, 0.15
Propensity score	−0.82	−1.11, −0.52
Study group (iCCM+) × visit week	−0.01	−0.01 – 0.00
Visit week × WHZ at admission	−0.02	−0.02, −0.02
Study group (iCCM+) × WHZ at admission	0.08	−0.02, 0.18
Random effects		
σ^2^		0.04
τ_00 id_		0.62
ICC		0.94
*N*_id_		1000
Observations		4999
Marginal *R*^2^/conditional *R*^2^		0.795/0.987

The model included a random intercept for each child to account for within-subject correlation from repeated weekly measurements. Fixed effects included study group, visit week, admission MUAC, admission WHZ, child sex, child age at admission, propensity score, and prespecified interaction terms. Continuous covariates were mean centered. Reference category for study group: OTP; reference category for sex: female.

Abbreviations: *β*, regression coefficient; CI, confidence interval; ICC, intraclass correlation coefficient, ICCM+, integrated community case management; MUAC, mid-upper arm circumference; OTP, outpatient therapeutic program; WHZ, weight-for-height Z-score.

Higher baseline WHZ (*β* = 0.34, *P* < 0.001), MUAC (*β* = 0.05, *P* < 0.001), and older age at admission (*β* = 0.15, *P* < 0.001) were associated with higher weight over follow-up. The interaction between visit week and admission WHZ was negative (*β* = –0.02, 95% CI: –0.02, 0.02, *P* < 0.001), indicating that children with higher WHZ at admission experienced smaller incremental increases in weight over time. Sensitivity analyses using propensity score–matched samples yielded consistent results (Supplemental Table 4).

### Time-to-recovery

**FIGURE 4:**
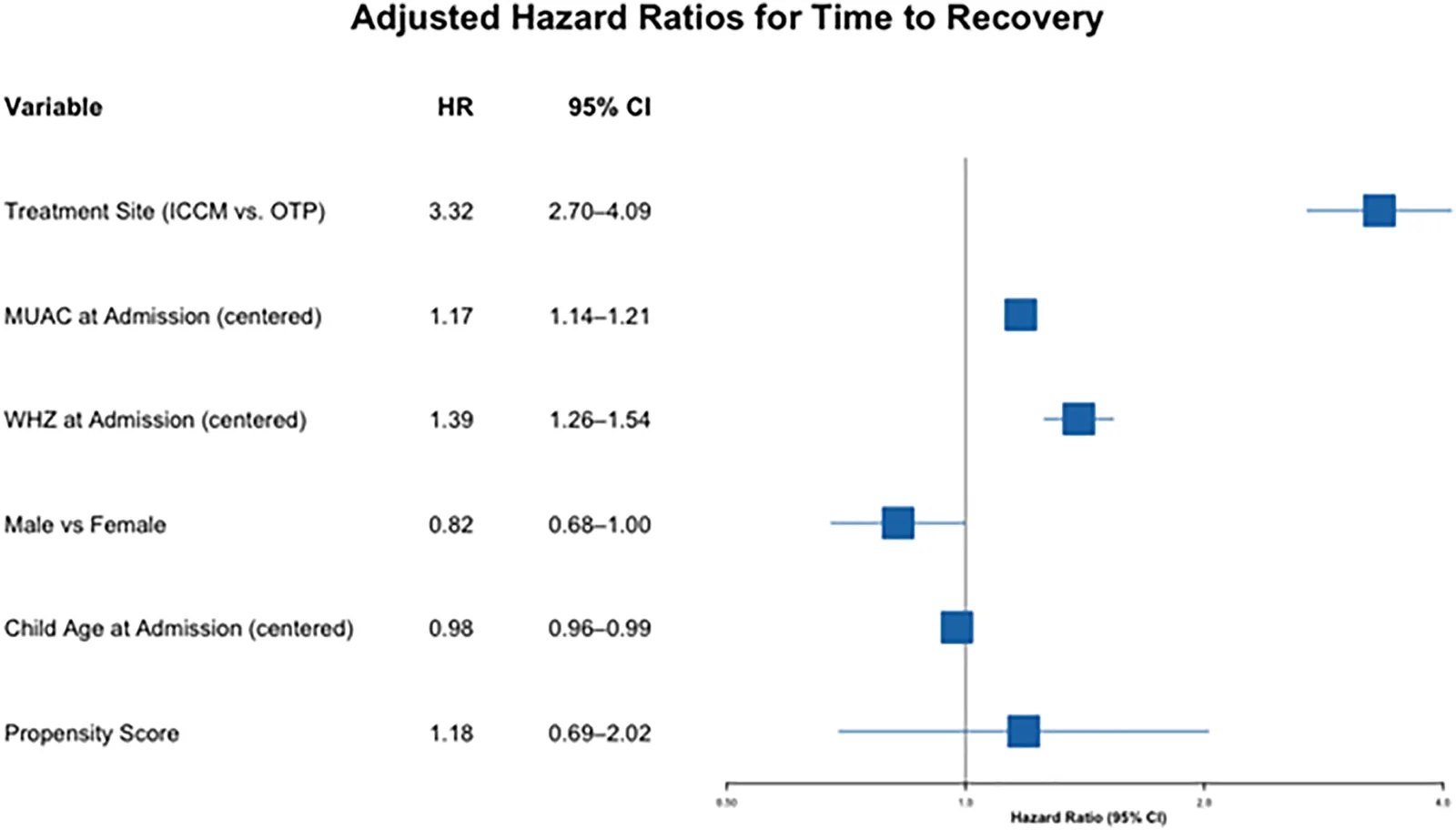
Forest plot showing adjusted hazard ratios from a Cox proportional hazards model for time-to-recovery among children with severe acute malnutrition in iCCM+ vs. OTP, Jowhar District, Somalia, January–May 2024. Hazard ratios > 1 indicate faster recovery. The model was adjusted for baseline MUAC (mean centered), baseline WHZ (mean centered), sex, age at admission (mean centered), and propensity score. Reference category for study group: OTP; reference category for sex: female. aHR, adjusted hazard ratio; CI, confidence interval; iCCM+, integrated community case management; MUAC, mid-upper arm circumference; OTP, outpatient therapeutic program; WHZ, weight-for-height Z-score.

Cox proportional hazards model (Figure 4) showed children in the iCCM+ group recovered faster than those in OTP, even after adjusting for baseline nutritional status, age, sex, and propensity score [adjusted hazard ratio (aHR) = 3.32, 95% CI: 2.70, 4.09; *P* < 0.001]. Higher admission MUAC and WHZ were associated with faster recovery. Each mm gain in MUAC was associated with a 17% increase in the hazard of recovery (aHR = 1.17, 95% CI: 1.14, 1.21; *P* < 0.001), and each unit gain in WHZ was associated with a 39% increase in hazard (aHR = 1.39, 95% CI: 1.26, 1.54; *P* < 0.001).

Sex was marginally associated with recovery time: males had an 18% lower hazard of recovery compared with females (aHR = 0.82, 95% CI: 0.68, 1.00; *P* = 0.05). Older admission age was significantly associated with slower recovery, with each month above the mean age associated with a 2% reduction in the hazard of recovery (aHR = 0.98, 95% CI: 0.96, 0.99; *P* < 0.001), indicating younger children recovered faster.

**FIGURE 5:**
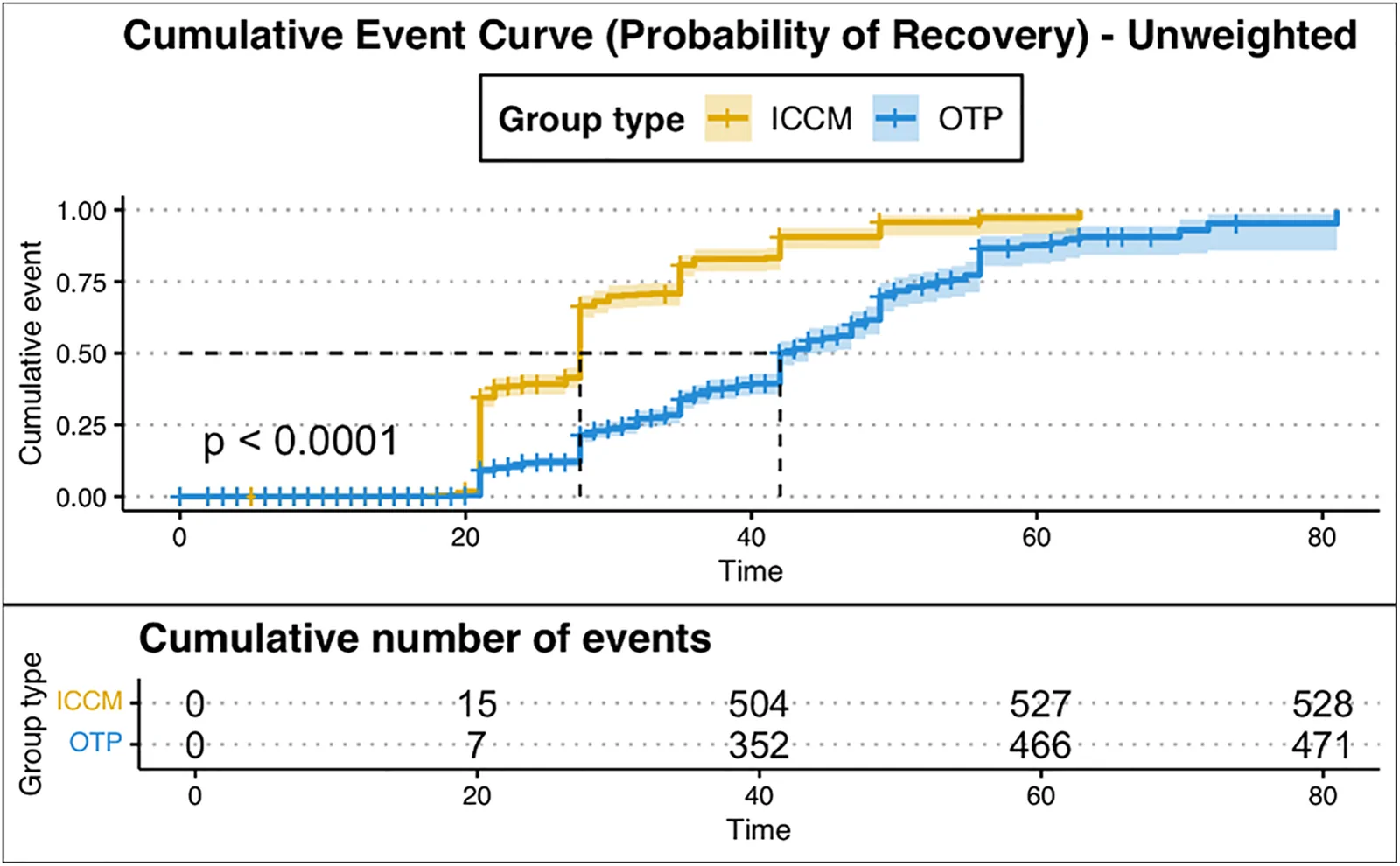
Kaplan–Meier cumulative recovery curves for children with severe acute malnutrition enrolled in iCCM+ and OTP groups, Jowhar District, Somalia, January–May 2024. Time is measured in days from admission. The event is defined as reaching moderate acute malnutrition criteria for ≥1 visit (intention-to-treat analysis). Children still in treatment at the study end date were censored (tick marks). Shaded bands represent 95% confidence intervals. Log-rank test *P* value is reported. iCCM+, integrated community case management; OTP, outpatient therapeutic program.

The cumulative recovery curve (Figure 5) showed a steeper early recovery trajectory in iCCM+, with over half of the children recovering by day 30. OTP recovery was slower, extending longer. At discharge, cumulative recovery proportions converged across groups, but time-to-recovery remained significantly shorter in iCCM+.

## Discussion

This study contributes to the growing body of evidence on treatment of uncomplicated SAM using the iCCM+ approach and, to our knowledge, is the first conducted in Somalia. Children in OTP presented with more severe anthropometric deficits, falling into the lowest MUAC and WHZ categories, likely due to daily screening in iCCM+ villages compared with monthly screening in OTP catchment areas. Similar findings have been seen in CHW-led screening results in earlier AM identification and admission [1,32]. Delayed SAM identification and initiation of treatment increase nutrition-related morbidity, mortality, and inpatient care, underscoring the value of expanding CHW screening coverage [33].

Depending on the definition of recovery, the iCCM+ group achieved comparable or better recovery outcomes than OTP. When recovery was defined more strictly according to the protocol as meeting discharge criteria at 2 consecutive visits, recovery was comparable across groups (89% in iCCM+ compared with 91% in OTP). When recovery was defined more broadly as meeting discharge criteria at least once, a higher proportion of children in the iCCM+ group recovered than in the OTP group (100% compared with 94%). Missed visits were more common in OTP, suggesting better attendance under iCCM+. LoS was 7 d shorter in iCCM+, likely reflecting both higher anthropometric status at admission and improved adherence facilitated by the proximity of services [34]. Importantly, neither group recorded defaulters or nonresponders, which might reflect the intensive supervision and data quality checks provided to both groups during the study. The comparable recovery and absence of negative outcomes suggest that, with adequate supervision and support, CHW-led care can be delivered safely and effectively.

Children in OTP had lower MUAC and WHZ scores and subsequently showed greater gains in both measures than those in iCCM+. Lower MUAC and WHZ scores at admission were associated with increased weight gain during treatment, which is in line with research showing children with poorer nutritional status at admission experience faster catch-up growth [35,36]. Weight trajectories also varied by study group: initial differences were minimal, but as treatment progressed, OTP-treated children gained weight more rapidly. This may reflect enhanced counseling or targeted support from OTP staff in response to slower early progress, as well as a more severe nutritional status [37,38].

This study has several strengths. Propensity score adjustment helped control baseline differences between groups, reducing bias and strengthening causal inference. Additionally, the study was conducted in a real-world implementation setting, making the results highly applicable to CMAM programs. Furthermore, iCCM+ staff determined both MUAC and WHZ, expanding the scope of the traditional MUAC-only CHW treatment model.

However, some limitations must be considered. Despite statistical adjustment, participants were not randomly assigned, and residual confounding may persist. In addition, 118 children were excluded because they did not meet study eligibility criteria or had missing or implausible anthropometric data. Although the overall exclusion proportion was similar between groups, incorrect eligibility criteria were more common in OTP, whereas implausible anthropometric data were more common in iCCM+. Missingness of data due to missing or implausible values was unlikely to be completely at random and may reasonably be considered compatible with a missing at random assumption. This exclusion may have created some selection bias. In addition, based on the directive issued by the Somali Nutrition cluster, WHZ and MUAC discharge criteria did not adhere to the global WHO discharge criteria. As a result, analytically estimated recovery may not fully align with programmatic recovery as observed in practice, which should be considered when interpreting differences between study findings and routine program outcomes. Furthermore, some children were discharged after meeting the discharge criteria at a single visit rather than at 2 consecutive visits, as required by the Somali integrated management of acute malnutrition (IMAM) protocol. This should be taken into account when interpreting the LoS. Additionally, relapse and sustained nutritional recovery were not evaluated. Finally, although the absence of defaulters and nonresponders is positive, it may reflect the intensive supervision provided during this study; defaulting and nonresponse could be higher under routine program conditions. At the same time, implementation under real-world conditions in a hard-to-reach area setting strengthens the applicability of the findings for other humanitarian contexts. However, if this approach is implemented at scale, the intensity of supervision may be lower, potentially impacting treatment outcomes. Future studies with longer follow-up periods, assessments of operational feasibility, and implementation across diverse geographic and health system contexts will be important to determine whether this approach can be successfully replicated at scale.

In conclusion, this study supports the scale-up of iCCM+ in hard-to-reach areas of Somalia. ICCM+ contributed to earlier identification of AM, with a smaller proportion of children admitted in the lowest MUAC and WHZ categories, whereas achieving treatment outcomes comparable to OTP. Additionally, iCCM+ was associated with a shorter LoS, suggesting potential benefits in efficacy and reduced burden on caregivers and health systems. Given its demonstrated feasibility and comparable effectiveness, iCCM+ offers a promising strategy for delivering SAM treatment in areas with limited access to OTP services. Optimizing the use of available resources is critical, and iCCM+ may be key to improving treatment in resource-constrained settings.

## Author contributions

The authors’ responsibilities were as follows – IB, JM, NMN: led on the funding acquisition; IB, JM, SK, NMN: designed the research; NMN, GAG, ZDA, AEM: conducted the research; IB, JM, SK: led the data analysis and wrote the initial paper; NMN, AEM, HV, GAG, ZDA, AM-B: critically reviewed and edited the manuscript; and all authors: read and approved the final manuscript.

## Data availability

Data described in the manuscript, code book, and analytic code will be made available on request pending application and approval.

## Disclaimer

The findings and conclusions in this report are those of the authors and do not necessarily represent the official position of the US Centers for Disease Control and Prevention nor the funder.

## Funding

The intervention was funded by United States Agency for International Development (USAID)–Bureau of Humanitarian Assistance, under award number 720BHA21GR00233. However, the research component did not receive external funding. USAID had no role in the design of the study; in the collection, analysis, and interpretation of data; or in writing the manuscript.

## Declaration of generative AI and AI-assisted technologies in the writing process

During the preparation of this work, the authors used ChatGPT 4o to assist in developing portions of the R code used for data analysis and for copy editing. After using this tool, the authors reviewed and edited the content as needed and take full responsibility for the content of the publication.

## Conflict of interest

IB reports financial support was provided by United States Agency for International Development (USAID)–Bureau for Humanitarian Assistance. The other authors report no conflicts of interest.
