# Supplementary material for: Integrated community case management plus compared with outpatient therapeutic programs: a quasi-experimental study among children with severe acute malnutrition in Jowhar, Somalia
Source: Am J Clin Nutr. 2026 Jun 9;124(2):101391. doi: 10.1016/j.ajcnut.2026.101391 (PMC13494071; doi:10.1016/j.ajcnut.2026.101391)
Supplement: Supplementary file 1 [file mmc1.docx]

**Supplemental Tables and Figures**

**Supplemental Figure 1. Distribution of propensity scores after matching**

***Supplemental Table 1.* Comparison of treatment outcomes among children with complete discharge outcomes enrolled in iCCM+ or OTP approach for severe acute malnutrition, Somalia, per protocol analysis**

| **Variable** | **N** | **Overall**  N = 1,183^1^ | **ICCM Site**  N = 545^1^ | **OTP site**  N = 638^1^ | **p-value^2^** |
| --- | --- | --- | --- | --- | --- |
| **Discharge outcome, n (%)** | 1,183 |  |  |  | <0.001 |
| *Censored / In-Treatment* |  | 186 (16%) | 19 (3%) | 167 (26%) |  |
| *Discharge Error - SAM Referred to TSFP* |  | 24 (2%) | 12 (2%) | 12 (2%) |  |
| *Recovered / Transferred to TSFP* |  | 973 (82%) | 514 (94%) | 459 (72%) |  |
| **Recovered with at least one visit among all children, n (%)** | 1,183 | 997 (84%) | 526 (97%) | 471 (74%) | <0.001 |
| **Recovered with two consecutive visits among non-censored, n (%)** | 947 | 856 (90%) | 460 (89%) | 396 (91%) | 0.308 |
| *Unknown* |  | 236 | 31 | 205 |  |
| **Length of stay (days)** | 1,152 | 28 (21, 35) | 21 (21, 28) | 28 (21, 42) | <0.001 |
| *Unknown* |  | 31 | 1 | 30 |  |
| **Cumulative RUTF (# of sachets) received** | 1,149 | 80 (60 100) | 75 (60, 90) | 85 (65, 120) | <0.001 |
| *Unknown* |  | 34 | 3 | 31 |  |
| **WHZ at discharge** | 1,152 | -1.60 (-2.32, -0.82) | -1.74 (-2.38, -0.96) | -1.44 (-2.25, -0.68) | <0.001 |
| *Unknown* |  | 31 | 1 | 30 |  |
| **Total weight gain (kg)** | 1,152 | 0.70 (0.50, 1.00) | 0.60 (0.40, 0.80) | 0.80 (0.60, 1.20) | <0.001 |
| *Unknown* |  | 31 | 1 | 30 |  |
| **Weight gain per day (kg/d)** | 1,152 | 0.03 (0.02, 0.03) | 0.02 (0.02, 0.03) | 0.03 (0.02, 0.03) | <0.001 |
| *Unknown* |  | 31 | 1 | 30 |  |
| **MUAC at discharge (mm)** | 1,150 | 117 (116, 119) | 116 (116, 117) | 118 (117, 123) | <0.001 |
| *Unknown* |  | 33 | 1 | 32 |  |
| **Total MUAC gain (mm)** | 1,150 | 5 (3, 9) | 4 (3, 5) | 8 (5, 12) | <0.001 |
| *Unknown* |  | 33 | 1 | 32 |  |
| **MUAC gain per day (mm/day)** | 1,150 | 0.21 (0.14, 0.29) | 0.14 (0.11, 0.20) | 0.25 (0.20, 0.30) | <0.001 |
| *Unknown* |  | 33 | 1 | 32 |  |
| **WAZ at discharge** | 1,152 | -2.59 (-3.23, -1.86) | -2.71 (-3.36, -2.00) | -2.48 (-3.14, -1.74) | <0.001 |
| *Unknown* |  | 31 | 1 | 30 |  |
| **HAZ at discharge** | 1,152 | -2.88 (-3.95, -1.58) | -2.87 (-3.99, -1.56) | -2.89 (-3.91, -1.60) | 0.948 |
| *Unknown* |  | 31 | 1 | 30 |  |
| ^1^n (%); Median (Q1, Q3) | | | | | |
| ^2^Pearson's Chi-squared test; Wilcoxon rank sum test | | | | | |

***Supplemental Table 2. Missed follow-up visits during treatment by study cohorts***

|  | **OTP** | | | | | **iCCM+** | | | | | |
| --- | --- | --- | --- | --- | --- | --- | --- | --- | --- | --- | --- |
|  | **n** | **N** | | **%** | | **n** | | **N** | | **%** | |
| **No. of missed visits per scheduled treatment follow-up visit** |  |  | |  | |  | |  | |  | |
| Enrollment | 0 | 638 | | 0% | | 0 | | 545 | | 0% | |
| Visit 1 | 31 | 622 | | 5% | | 3 | | 545 | | 1% | |
| Visit 2 | 48 | 602 | | 8% | | 5 | | 543 | | 1% | |
| Visit 3 | 21 | 562 | | 4% | | 0 | | 531 | | 0% | |
| Visit 4 | 24 | 384 | | 6% | | 0 | | 220 | | 0% | |
| Visit 5 | 14 | 244 | | 6% | | 0 | | 63 | | 2% | |
| Visit 6 | 7 | 152 | | 5% | | 0 | | 24 | | 0% | |
| Visit 7 | 3 | 81 | | 4% | | 0 | | 8 | | 0% | |
| Visit 8 | 0 | 27 | | 0% | | 0 | | 2 | | 0% | |
| Visit 9 | 2 | 8 | | 25% | | 0 | | 1 | | 0% | |
| Visit 10 | 0 | 4 | | 0% | | - | | - | | - | |
| Visit 11 | 0 | 1 | | 0% | | - | | - | | - | |
| Visit 12 | 0 | 1 | | 0% | | - | | - | | - | |
| All Visits | 150 | 3325 | | 5% | | 8 | | 2482 | | <1% | |
| **No. of children who missed scheduled treatment follow-up visits** |  |  | |  | |  | |  | |  | |
| No visits missed | 508 | 638 | | 80% | | 537 | | 545 | | 99% | |
| One visit missed | 118 | 638 | | 18% | | 8 | | 545 | | 1% | |
| Two visits missed | 6 | 638 | | 1% | | 0 | | 545 | | 0% | |
| Three or more visits missed | 6 | 638 | | 1% | | 0 | | 545 | | 0% | |
| **No. of children who missed consecutive scheduled treatment follow-up visits** | | |  | |  | |  | |  | |  |
| No consecutive missed visits | 632 | 638 | | 99% | | 545 | | 545 | | 100% | |
| One consecutive missed visit | 1 | 638 | | <1% | | 0 | | 545 | | 0% | |
| Two consecutive missed visits | 4 | 638 | | 1% | | 0 | | 545 | | 0% | |
| Three or more consecutive missed visits | 1 | 638 | | <1% | | 0 | | 545 | | 0% | |

*ICCM+ = integrated community case management*

***Supplemental Table 3. Interpolated vs. non-interpolated value comparison by study cohorts***

|  | **OTP Treatment** | | | | | | | | **iCCM+ Treatment** | | | | | | | |
| --- | --- | --- | --- | --- | --- | --- | --- | --- | --- | --- | --- | --- | --- | --- | --- | --- |
|  | **Missing Values** | | | **Non-Interpolated Values** | | **All Values** | | **p-value** | **Missing Values** | | | **Non-Interpolated Values** | | **All Values** | | **p-value** |
|  | **n** | **N** | **%** | **n** | **mean (SD)** | **n** | **mean (SD)** |  | **n** | **N** | **%** | **n** | **mean (SD)** | **n** | **mean (SD)** |  |
| **Overall** |  |  |  |  |  |  |  |  |  |  |  |  |  |  |  |  |
| MUAC (mm) | 150 | 3325 | 5% | 3175 | 115.1 (5.1) | 3325 | 115.1 (5.1) | 0.99 | 8 | 2482 | <1% | 2474 | 114.2 (3.2) | 2482 | 114.2 (3.2) | 1.00 |
| Weight (kg) | 151 | 3326 | 5% | 3175 | 7.7 (1.8) | 3326 | 7.7 (1.8) | 0.99 | 8 | 2482 | <1% | 2474 | 7.3 (1.7) | 2482 | 7.3 (1.7) | 1.00 |
| Height (cm) | 151 | 3326 | 5% | 3175 | 74.3 (11.3) | 3326 | 74.3 (11.3) | 1.00 | 8 | 2482 | <1% | 2474 | 71.8 (9.5) | 2482 | 71.8 (9.5) | 1.00 |

^I^*All values include both non-interpolated values plus interpolated values*

*^*^Student t-test*

***Supplemental Table 4***: ***Mixed model treatment effect on weight gain during treatment using propensity scores***

|  | **Propensity score matched - Mixed model treatment effect on weight during treatment** | | |
| --- | --- | --- | --- |
| *Predictors* | *Estimates* | *CI* | *p* |
| (Intercept) | 6.93 | 6.80 – 7.06 | **<0.001** |
| Study arm [iCCM+] | 0.01 | -0.14 – 0.15 | 0.935 |
| Visit week | 0.18 | 0.18 – 0.19 | **<0.001** |
| MUAC at admission (mean-centered) | 0.04 | 0.02 – 0.06 | **<0.001** |
| WHZ at admission (mean-centered) | 0.32 | 0.20 – 0.44 | **<0.001** |
| Child sex [male] | -0.21 | -0.34 – -0.08 | **0.002** |
| Child age (months) (mean-centered) | 0.14 | 0.14 – 0.15 | **<0.001** |
| Study arm [iCCM+] × Visit week | -0.00 | -0.01 – 0.01 | 0.488 |
| Visit week × WHZ at admission (mean-centered) | -0.03 | -0.03 – -0.02 | **<0.001** |
| Study arm [iCCM+] × WHZ at admission (mean-centered) | 0.12 | -0.02 – 0.26 | 0.093 |
| **Random Effects** | | | |
| σ^2^ | 0.05 | | |
| τ_00_ _id_ | 0.61 | | |
| ICC | 0.93 | | |
| N _id_ | 599 | | |
| Observations | 2906 | | |
| Marginal R^2^ / Conditional R^2^ | 0.767 / 0.983 | | |
